# Efficacy of Dupilumab in Treating Trachyonychia and Other Nail Dystrophies: A Systematic Review of Case Reports

**DOI:** 10.7759/cureus.104807

**Published:** 2026-03-07

**Authors:** Shaden A Alhazmi, Shatha Albyali, Rawan Alanazi, Baraa S Alhejaili, Abdulaziz S Alqahtani, Raghad K Altowairqi, Rawan Mousa Altamimi, Naif M Alzahrani, Bashayr Alamoudi, Kadi A Alsweed, Razan Kurdi, Hala S Hajjaj, Rushdan Firdous, Lara M Samhan, Arwa O Al-Modayfer

**Affiliations:** 1 College of Medicine, Imam Mohammad Ibn Saud Islamic University, Riyadh, SAU; 2 College of Medicine, Alfaisal University, Riyadh, SAU; 3 College of Medicine, Dar Al Uloom University, Riyadh, SAU; 4 College of Medicine, Taibah University, Madinah, SAU; 5 College of Medicine, King Khalid University, Abha, SAU; 6 College of Medicine, King Abdulaziz University, Jeddah, SAU; 7 College of Medicine, King Saud University, Riyadh, SAU; 8 College of Medicine, King Saud bin Abdulaziz University for Health Sciences, Riyadh, SAU; 9 College of Pharmacy, Riyadh Elm University, Riyadh, SAU; 10 College of Medicine, Qassim University, Buraydah, SAU; 11 Department of Medicine and Surgery, Taibah University, Madinah, SAU; 12 College of Medicine, University of Jeddah, Jeddah, SAU; 13 Department of Dermatology, King Saud Medical City, Riyadh, SAU

**Keywords:** biologic therapy in nail disorders, dupilumab, inflammatory dermatoses and nails, nail dystrophies, nail growth recovery

## Abstract

Trachyonychia and related nail dystrophies are often associated with inflammatory skin diseases such as atopic dermatitis, psoriasis, and alopecia areata. Management remains difficult, as standard therapies frequently yield limited benefit. Dupilumab, an IL-4Rα antagonist, is effective for Th2-driven conditions, but its role in nail dystrophies has not been systematically assessed. This review evaluates the existing evidence on dupilumab for trachyonychia and related nail disorders.

A systematic search of PubMed, Wiley, Web of Science, MEDLINE, Google Scholar, and EBSCO was performed from inception to July 2025 with a string of specified keywords. Studies reporting patients with nail dystrophy treated with dupilumab were included. Two reviewers independently extracted data and assessed study quality using the Joanna Briggs Institute Critical Appraisal Checklist for Case Reports.

Eight case reports met the inclusion criteria. Trachyonychia, twenty-nail dystrophy, median canaliform dystrophy, and mixed onychodystrophies were represented. All reports described clinical improvement, with several achieving near-complete recovery. Time to improvement ranged from weeks to over a year, with pediatric patients often responding faster. Dupilumab was well tolerated, with no serious adverse effects reported. Concurrent therapies limited the attribution of outcomes solely to dupilumab.

Current evidence suggests dupilumab may improve nail dystrophies associated with Th2-mediated disease. However, evidence remains limited to isolated case reports. Larger controlled studies using standardized nail outcome measures are needed to confirm efficacy.

## Introduction and background

The nail apparatus (NA) is a specialized keratinized structure essential for protecting the distal phalanx, supporting fine motor function, and maintaining digital homeostasis. It comprises the nail plate and its supporting components, including the proximal and lateral nail folds, nail matrix, nail bed, and periungual region or hyponychium [1]. Owing to its complex architecture, the NA can be significantly affected by a wide spectrum of inflammatory dermatologic disorders. Chronic inflammatory conditions such as atopic dermatitis, psoriasis, lichen planus, and alopecia areata may cause periungual inflammation and subsequent nail dystrophy, underscoring the NA's susceptibility to immune-mediated skin diseases [2]. Among the various nail changes observed in these disorders, matrix-predominant inflammation with spongiosis and lymphocytic infiltration is particularly relevant, as it underlies several forms of inflammatory nail dystrophy and provides a mechanistic bridge to type 2-skewed immune responses [3].

Trachyonychia is an inflammatory disorder predominantly involving the nail matrix. Clinically, it presents with longitudinal ridging, diffuse pitting, and a roughened nail surface often described as "sandpaper nails" [4]. Although frequently idiopathic and more common in children, trachyonychia is also associated with several chronic inflammatory diseases, including psoriasis, lichen planus, atopic dermatitis, and alopecia areata [4]. Diagnosis is primarily clinical. When performed, histopathologic examination typically reveals spongiotic inflammation within the nail matrix [4]. Management is challenging: topical therapies are frequently ineffective, and intralesional triamcinolone injections, while sometimes attempted, are painful, invasive, and impractical for routine use [4]. Importantly, the combination of matrix-predominant spongiosis and its association with atopic dermatitis and other type 2-biased dermatoses positions trachyonychia as a clinically relevant model for exploring targeted Th2-modulating therapies at the level of the nail unit [5].

In addition to trachyonychia, this review considers other inflammatory nail dystrophies characterized by matrix or periungual involvement, including nail changes in atopic dermatitis (such as roughness, ridging, and pitting), nail psoriasis (pitting, onycholysis, subungual hyperkeratosis), nail lichen planus (longitudinal ridging, fissuring, and thinning), and alopecia areata-associated nail dystrophy (trachyonychia-like roughness and punctate depressions). These entities share overlapping clinical features rooted in chronic inflammation of the nail matrix and bed and often show histopathologic evidence of interface change or spongiotic/lichenoid inflammation, suggesting convergent pathogenic pathways. Because atopic dermatitis, many forms of alopecia areata-associated nail disease, and a subset of psoriatic and eczematous nail changes are driven at least in part by type 2 immune responses, they represent rational targets for therapies that block key Th2 cytokine pathways [3,5,6].

Dupilumab is a fully human monoclonal antibody that blocks the interleukin-4 receptor alpha (IL-4Rα) subunit, inhibiting both IL-4 and IL-13 signaling pathways central to type 2 inflammation [7,8]. By targeting IL-4Rα, dupilumab suppresses downstream chemokines, proinflammatory cytokines, and IgE, thereby dampening the broader Th2-mediated inflammatory cascade [7]. While its efficacy is well established for atopic dermatitis and other type 2 inflammatory conditions, emerging case reports suggest that dupilumab may also reduce inflammatory activity within the nail unit, enabling the gradual normalization of nail growth [3,8,9]. However, its therapeutic role in trachyonychia and other nail dystrophies remains insufficiently characterized, and no large-scale studies have evaluated nail-specific outcomes [3].

Given the absence of randomized controlled trials and the limited availability of nail-focused clinical data, this systematic review of case reports synthesizes the current evidence on dupilumab's potential benefits in trachyonychia and related nail dystrophies.

Within the framework of scarce nail-specific clinical literature, this systematic review consolidates existing evidence regarding dupilumab's potential efficacy in trachyonychia and associated nail dystrophies. Accordingly, the objective of this systematic review is to synthesize and critically appraise the available clinical evidence on dupilumab for trachyonychia and other inflammatory nail dystrophies, with a specific focus on nail-specific outcomes, associated underlying diseases, treatment regimens, and safety, in order to better inform clinicians about the potential role of Th2-targeted biologic therapy in the management of nail unit inflammation.

## Review

Methodology

Protocol and Study Registration

This review was registered with the International Prospective Register of Systematic Reviews (PROSPERO) (CRD420251103183) and conducted in accordance with the Preferred Reporting Items for Systematic Reviews and Meta-Analyses (PRISMA) reporting principles.

Search Strategy

A comprehensive search was performed in PubMed, Wiley, Web of Science, MEDLINE, Google Scholar, and EBSCO from database inception to July 2025. Search concepts combined disease terms, intervention, and mechanistic keywords using Boolean operators and truncation as appropriate. Keywords included the following: "dupilumab", "trachyonychia", "nail dystrophies", "nail matrix inflammation", "atopic dermatitis", "Th2-mediated conditions", "twenty-nail dystrophy", "median canaliform dystrophy", "onychodystrophy", "dupilumab efficacy", "IL-4 receptor antagonism", "nail growth recovery", "biologic therapy in nail disorders", "nail matrix regeneration", "dupilumab side effects", and "inflammatory dermatoses and nails".

Reference lists of included articles were hand-searched for additional studies. No language or date limits were applied at the database level. Google Scholar was used to identify grey literature and hard-to-index case reports.

Study Selection

Eligible studies were case reports or case series describing human patients with nail dystrophy (e.g., trachyonychia, twenty-nail dystrophy, median canaliform dystrophy, mixed onychodystrophies) treated with dupilumab, reporting any clinical nail outcome. All ages and comorbidity profiles were eligible.

**Figure 1:**
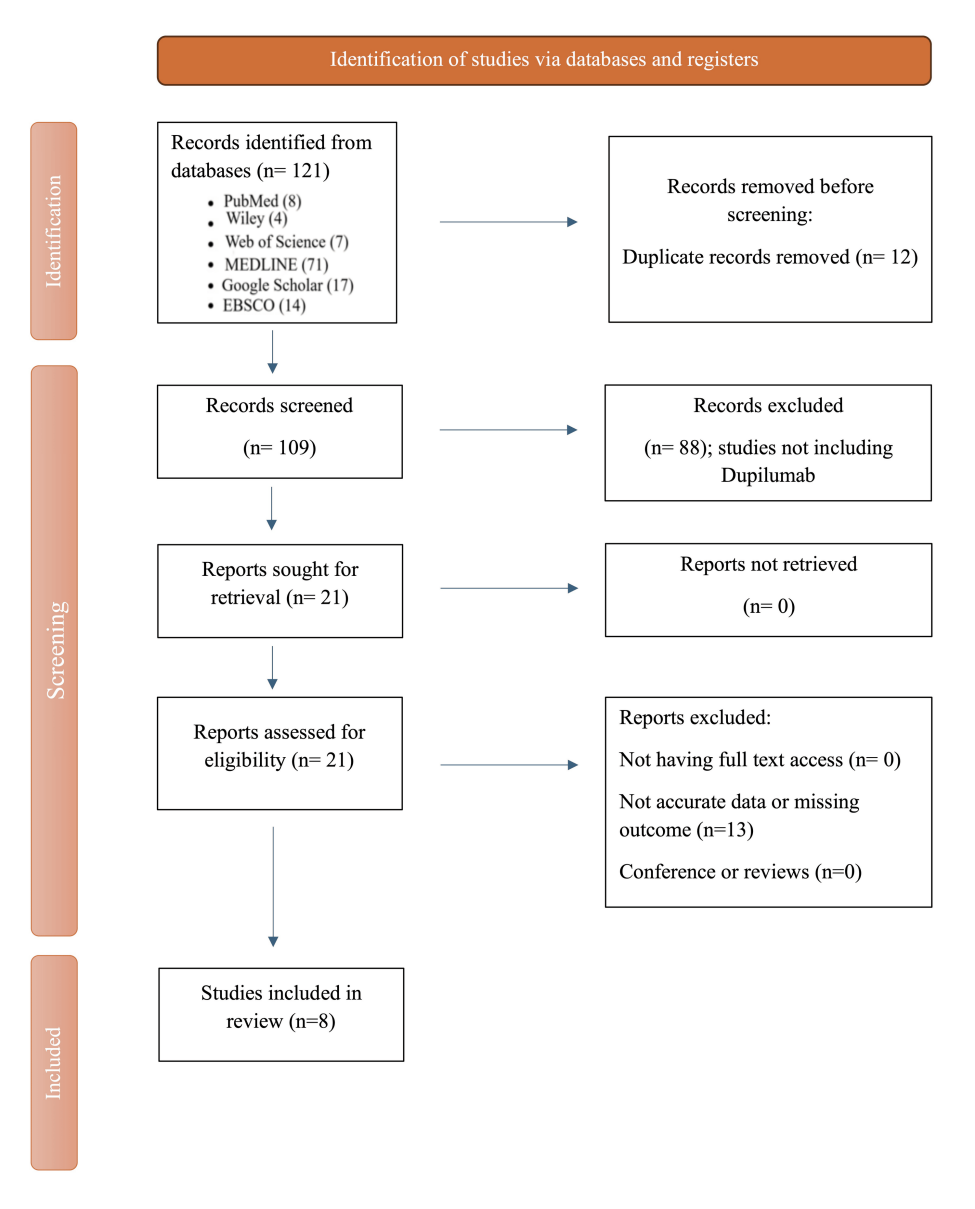
PRISMA checklist of studies' selection PRISMA: Preferred Reporting Items for Systematic Reviews and Meta-Analyses

Exclusions were the following: non-human studies, narrative reviews without primary cases, conference abstracts lacking outcome data, and reports without nail outcomes. Two reviewers independently screened titles/abstracts and then full texts; discrepancies were resolved by consensus with a third reviewer. Selection steps are summarized in the PRISMA flow diagram (refer to Figure 1).

Data Extraction

Two reviewers independently extracted data on publication details, study characteristics, patient demographics, diagnoses, dupilumab regimens, concurrent therapies, outcomes, and adverse effects. Discrepancies were resolved through discussion with a third reviewer.

Critical Appraisal

The Joanna Briggs Institute (JBI) Critical Appraisal Checklist for Case Reports was used by two reviewers independently to assess methodological quality across eight domains [10]. Disagreements were resolved through discussion with a third reviewer. While no studies were excluded based on their quality score, we used the JBI results to identify which cases provided the most complete clinical data.

Data Synthesis

Given the descriptive nature of the included studies and the absence of controlled or comparative data, a narrative data synthesis approach was adopted. Findings from the included case reports were summarized qualitatively rather than pooled quantitatively. Data were synthesized across studies by grouping cases according to the type of nail dystrophy, underlying dermatologic condition, dupilumab treatment regimen, time to clinical response, degree of nail improvement, and reported adverse events.

Results

Clinical Presentations of Nail Dystrophies

Eight published single-patient case reports evaluating dupilumab were included (Table 1).

**Table 1:** Clinical and treatment characteristics of the included case reports The table presents a summary of clinical characteristics, nail findings, and reported outcomes of the included case reports evaluating dupilumab. Nail outcomes and timelines are reported as described by the original authors.

Study (author, year)	Country	Age/sex	Underlying condition	Type of nail dystrophy	Dupilumab regimen	Time to initial nail changes	Nail outcome
Li et al., 2025 [2]	China	40/F	Atopic dermatitis	Twenty-nail dystrophy (trachyonychia)	600 mg loading; 300 mg every 2 weeks → every 3 weeks	2 weeks (symptoms), 2 months (growth), 5 months	Near-complete recovery (except thumbs)
Chung et al., 2025 [4]	USA	5/M	Atopic dermatitis, alopecia areata	Trachyonychia	300 mg subcutaneously every 4 weeks	~3 months	Marked improvement in nail pitting and ridging
Johar et al., 2023 [7]	Saudi Arabia	6/F	DOCK8-deficiency hyper-IgE syndrome	Onychodystrophy with subungual keratosis	200 mg SC every 4 weeks	~3 months	Healthy nail regrowth
Özkaya et al., 2023 [11]	Turkey	22/M	Netherton syndrome	Thickened, ridged nails	600 mg loading; 300 mg every 2 weeks	~32 weeks	Improvement in nail thickness and texture
Navarro-Triviño et al., 2021 [12]	Spain	61/M	Severe atopic dermatitis	Mixed onychodystrophy	Regimen not specified	~3 months	Partial improvement
Zubek and Vesely, 2021 [3]	USA	60/F	Atopic dermatitis	Mixed nail dystrophy	300 mg every 2 weeks	~15 months	Gradual improvement after initial worsening
Gruber et al., 2021 [13]	Austria	8/M	Trichothiodystrophy	Dystrophic nails	200mg every 2 weeks	~12 months	Thicker, healthier nail growth
Giura et al., 2020 [14]	Italy	Adult/F	Atopic dermatitis	Median canaliform nail dystrophy	Initial dose of 600 mg, followed by 300 mg, every 2 weeks	~4 months	Complete remission

Trachyonychia was the most frequently reported presentation, manifesting as either the classic twenty-nail dystrophy or more localized [2,4]. Other reported nail conditions included median canaliform dystrophy [14] and dystrophic nails associated with trichothiodystrophy [13], both secondary to systemic disease. Mixed or complex dystrophies are characterized by onychorrhexis, ridging, hyperkeratosis, or onycholysis [12,3]. Behavioral and inflammatory etiologies were also represented, such as dystrophy accompanied by subungual keratosis and nail thickening [7]. Thickened and ridged nails were also reported in the context of Netherton syndrome [11].

Across all reports, nail findings were described qualitatively, based on clinical observation rather than standardized nail-specific severity scoring tools (Table 2).

**Table 2:** Reported comorbidities across the included case reports

Study (author, year)	Atopic dermatitis	Alopecia areata	Genetic/syndromic disease	Other
Li et al., 2025 [2]	Yes	No	No	-
Chung et al., 2025 [4]	Yes	Yes	No	-
Johar et al., 2023 [7]	Yes	No	DOCK8-deficiency	Recurrent eczema herpeticum
Özkaya et al., 2023 [11]	Yes	No	Netherton syndrome	-
Navarro-Triviño et al., 2021 [12]	Yes	No	No	-
Zubek and Vesely, 2021 [3]	Yes	No	No	-
Gruber et al., 2021 [13]	Yes	No	Trichothiodystrophy	-
Giura et al., 2020 [14]	Yes	No	No	-

Treatment Outcomes With Dupilumab

All eight case reports described clinical resolution in nail dystrophy following dupilumab therapy, though the extent and speed of response varied. Some patients, particularly those with trachyonychia and median canaliform dystrophy, showed near-complete or complete resolution within 3-5 months [2,4,14]. Others show more gradual changes over the course of 12-15 months, such as thicker, healthier nail growth, especially those with other genetic conditions [7,3,11,12,13].

Initial nail changes were reported within weeks to months, but more significant nail normalization typically needed longer follow-up.

Safety and Tolerability

Dupilumab was reported to be generally well tolerated across the included studies, with no significant systemic or treatment-limiting adverse effects reported.

Methodological Characteristics of the Included Reports

Each one of the studies had a different follow-up duration. Clinical observation and photographic documentation were the main methods used to evaluate outcomes rather than standardized nail-specific severity scoring tools. Information about prior or concurrent treatments was inconsistent; some patients only received dupilumab, while others received other therapies but were unsuccessful. This heterogeneity makes it difficult to compare results between cases (Table 3).

**Table 3:** Prior and concomitant treatments reported in the included case reports

Study (author, year)	Prior treatments	Concomitant treatments with dupilumab
Li et al., 2025 [2]	Antihistamines, topical halometasone cream (a corticosteroid), pimecrolimus ointment (a topical calcineurin inhibitor), oral corticosteroid treatment	None reported
Chung et al., 2025 [4]	For alopecia areata: topical steroids, topical minoxidil, fexofenadine, and a 12-week course of pulsed dexamethasone. For atopic dermatitis: topical steroids, specifically triamcinolone 0.1% ointment and mometasone 0.1% ointment	The patient was still using topical steroids. However, by the three-month follow-up, atopic dermatitis had improved, so topical steroid treatment was no longer required
Johar et al., 2023 [7]	Monthly intravenous immunoglobulin (IVIG) transfusions. Intravenous (IV) acyclovir, antibiotics, topical steroids	None reported
Özkaya et al., 2023 [11]	Cyclosporine A (initial phase)	Cyclosporine A (first 32 weeks)
Navarro-Triviño et al., 2021 [12]	Methotrexate, cyclosporin, alitretinoin, etanercept (a TNF-alpha inhibitor), adalimumab (a TNF-alpha inhibitor)	None reported
Zubek and Vesely, 2021 [3]	Not reported	None reported
Gruber et al., 2021 [13]	Topical corticosteroids, topical calcineurin inhibitors	Topical glucocorticoids, antihistamines
Giura et al., 2020 [14]	Topical steroids, systemic steroids, cyclosporine	None reported

Risk of Bias

A summary of the methodological quality of the eight case reports is given in Table 4. The highest possible score of 8/8 was given to three of the studies, indicating that the reports were generally of excellent quality. The remaining studies scored between 6/8 and 7/8, primarily due to insufficient reporting on adverse events (Q7) or post-intervention clinical status (Q6). In the context of this review, all of the studies were deemed to have acceptable methodological quality; however, none of them had reporting limitations.

**Table 4:** Joanna Briggs Institute (JBI) Critical Appraisal Checklist for Case Reports Q1: demographics; Q2: history/timeline; Q3: current clinical condition; Q4: diagnostic tests; Q5: interventions; Q6: post-intervention clinical condition; Q7: adverse events; Q8: takeaway lessons Articles with scores 1 and 2 were classified as low quality, 3-4 moderate quality, and 5-8 high quality.

Study (author, year)	Q1	Q2	Q3	Q4	Q5	Q6	Q7	Q8	Score
Li et al., 2025 [2]	Yes	Yes	Yes	Yes	Yes	Yes	No	Yes	7/8
Zubek and Vesely, 2021 [3]	Yes	Yes	Yes	Yes	Yes	Yes	Yes	Yes	8/8
Chung et al., 2025 [4]	Yes	Yes	Yes	Yes	Yes	Yes	Yes	Yes	8/8
Johar et al., 2023 [7]	Yes	Yes	Yes	Yes	Yes	Yes	No	Yes	7/8
Özkaya et al., 2023 [11]	Yes	Yes	Yes	Yes	Yes	Yes	Yes	Yes	8/8
Navarro-Triviño et al., 2021 [12]	Yes	Yes	Yes	Yes	No	Yes	No	Yes	6/8
Gruber et al., 2021 [13]	Yes	Yes	Yes	Yes	Yes	Yes	Yes	Yes	8/8
Giura et al., 2020 [14]	No	Yes	Yes	Yes	Yes	Yes	No	Yes	6/8

Discussion

Nails are dynamic keratinized structures whose growth and appearance often reflect underlying systemic or dermatologic conditions [15]. Their growth rate differs between fingernails and toenails, and disturbances may arise from trauma, infections, systemic diseases, or inflammatory dermatoses such as psoriasis and lichen planus [15-19]. Atopic dermatitis, a chronic inflammatory disease characterized by epidermal barrier dysfunction, is also associated with persistent hand and foot dermatitis and may involve the nail matrix, producing findings such as trachyonychia, pitting, and melanonychia [20-22]. Trachyonychia is primarily diagnosed clinically, with biopsies, when performed, showing spongiotic inflammation of the nail matrix [4,23]. Because Th2-driven inflammation is central to atopic dermatitis and related conditions, this review explored the potential of dupilumab, an IL‑4Rα antagonist, to improve nail dystrophies related to Th2-mediated disease.

Across the included case reports, dupilumab consistently improved a wide spectrum of nail dystrophies, particularly those associated with atopic dermatitis or other inflammatory conditions. Several cases demonstrated rapid and meaningful responses, such as near-complete improvement within five months in twenty-nail dystrophy or full resolution of median canaliform dystrophy within four months [2,14]. Pediatric patients with inflammatory or syndromic nail disease also showed notable improvements, including healthy regrowth or normalization over 3-12 months [4,7,13]. Other reports described partial improvement in mixed dystrophies or onychotillomania-associated changes, often requiring longer durations, up to 15 months, for substantial benefit [1,12]. Overall, these findings support the hypothesis that IL‑4/IL‑13 blockade reduces nail matrix inflammation, allowing the gradual restoration of normal nail growth.

Time to improvement varied considerably, ranging from early symptomatic relief within weeks to complete clinical resolution over more than a year. Early benefits, such as reduced pruritus or visible nail regrowth, were often noted within weeks, while complete recovery required longer periods consistent with slow nail growth rates (1-2 mm per month) [2,11]. Pediatric patients generally responded more quickly, whereas adults with chronic or complex dystrophies required prolonged treatment [3,4,7,12,13]. Findings from other studies on dupilumab in atopic dermatitis, including rapid improvement in cutaneous symptoms, support the early symptomatic improvements observed [2,11,14,24].

Interpretation of outcomes is limited by the frequent use of concurrent therapies, such as topical corticosteroids or immunomodulators, which obscure the direct contribution of dupilumab. Most cases followed standard atopic dermatitis dosing regimens, including a 600 mg loading dose followed by 300 mg every 2-4 weeks, with pediatric patients receiving weight-adjusted dosing (e.g., 200 mg every 2-4 weeks) [2,7,11,13,14]. No clear association was observed between dose intensity and time to improvement, suggesting that baseline disease severity and patient-specific factors likely play greater roles in determining response.

Dupilumab maintained a favorable safety profile across cases, with no serious adverse events reported [24]. Mild injection-site reactions or conjunctivitis, commonly observed in larger atopic dermatitis cohorts, were infrequent [24]. A large retrospective analysis of over 900 atopic dermatitis patients similarly reported no significant nail-related adverse effects, aligning with the benign safety findings in this review [25]. Although rare reports of transient nail worsening exist, these effects typically resolve with ongoing therapy [3].

Overall, the consistent temporal relationship between dupilumab initiation and nail improvement across diverse presentations suggests a therapeutic role for IL‑4/IL‑13 blockade in nail matrix inflammation. These findings expand the potential scope of dupilumab beyond its established dermatologic and respiratory indications. However, available evidence remains limited by reliance on single-patient reports, lack of objective nail scoring tools, and inconsistent documentation of disease course and response timelines.

Future research should include controlled studies with standardized outcome measures, clearly defined nail involvement, and objective and patient-reported endpoints. Further work is needed to clarify predictors of response, optimal dosing strategies, and long-term effects on nail unit biology.

## Conclusions

Available evidence from case reports suggests that dupilumab may be associated with improvement in nail dystrophies in patients with atopic dermatitis, with several reports describing partial to complete recovery over time. However, these observations remain exploratory due to the absence of controlled studies, small sample sizes, lack of standardized nail assessment tools, and potential confounding from concomitant therapies. Current evidence is limited to atopic dermatitis, and broader applicability cannot be assumed. Well-designed prospective studies incorporating objective and standardized nail outcome measures are needed to clarify the efficacy and therapeutic role of dupilumab in nail dystrophy associated with atopic dermatitis.
